# Inhaled budesonide and beclomethasone for the prevention and treatment of bronchopulmonary dysplasia in very preterm infants: a prospective randomized controlled trial

**DOI:** 10.3389/fped.2026.1818492

**Published:** 2026-06-04

**Authors:** Bingjie Wang, Siyuan Xu, Zheng Li, Kaidong Yang, Nuo Xu, Di Huang, Dandan Zhao, Bo Yang, Xiangyu Gao

**Affiliations:** Department of Neonatology, Xuzhou Clinical College of Xuzhou Medical University (Xuzhou Central Hospital), Xuzhou, China

**Keywords:** beclomethasone, bronchopulmonary dysplasia, budesonide, infant, premature, inhalation

## Abstract

**Background:**

To investigate the clinical efficacy and safety of inhaled budesonide and beclomethasone for the prevention and treatment of bronchopulmonary dysplasia (BPD) in very preterm infants.

**Methods:**

From October 2024 to November 2025, very preterm infants at high risk of BPD, aged >7 days and still requiring non-invasive respiratory support, who were admitted to the Neonatal Intensive Care Unit of Xuzhou Central Hospital, were selected and randomly divided into three groups. The control group received inhaled normal saline at a dose of 1 mL per administration, once every 12 h (q 12 h); the budesonide group received inhaled budesonide at a dose of 0.5 mg per administration, q 12 h; the beclomethasone group received inhaled beclomethasone at a dose of 0.4 mg per administration, q 12 h. Inhalation treatment was continued in all three groups until respiratory support was no longer needed. The total duration of respiratory support, the incidence and severity of BPD, the incidence of adverse outcomes, and inflammatory markers were compared among the three groups. Multivariate Logistic regression analysis was used to identify the independent risk factors and protective factors for BPD in very preterm infants.

**Results:**

Finally, 37 cases were enrolled in the control group, 41 cases in the budesonide group, and 40 cases in the beclomethasone group. The total duration of respiratory support and length of hospital stay in the budesonide group (36.4 ± 14.9 d, 39.5 ± 10.1 d) and the beclomethasone group (35.6 ± 15.1 d, 39.8 ± 11.0 d) were shorter than those in the control group (43.5 ± 13.5 d, 50.4 ± 8.5 d), with statistically significant differences (*P* < 0.05). There were no statistically significant differences among the three groups in terms of the incidence of BPD, the incidence of mild and moderate-severe BPD, the mean values of fraction of inspired oxygen and oxygen saturation index within 48 h after initiation of treatment, the incidence of late-onset sepsis, stage Ⅱ–Ⅲ necrotizing enterocolitis, grade Ⅱ–Ⅳ intraventricular hemorrhage, the incidence of treatment-requiring retinopathy of prematurity at initial screening, the volume of packed red blood cell transfusion, hemodynamically significant patent ductus arteriosus at discharge, and hospitalization costs (*P* > 0.05). On the 14th day after treatment initiation, the levels of interleukin-8 were lower and the levels of interleukin-10 were higher in both the budesonide group and the beclomethasone group than those in the control group, with statistically significant differences (*P* < 0.05). There were no statistically significant differences in any of the indicators between the budesonide group and the beclomethasone group (*P* > 0.05). Younger gestational age and lower birth weight were identified as independent risk factors for BPD in very preterm infants, whereas inhaled budesonide or beclomethasone administration after the first week of life served as independent protective factors against BPD.

**Conclusions:**

Inhaled budesonide and beclomethasone after the first week of life can shorten the total duration of respiratory support in very preterm infants at high risk of BPD, aged > 7 days and still require non-invasive respiratory support, without increasing the incidence of adverse outcomes, demonstrating high safety. However, inhaled budesonide and beclomethasone do not show significant advantages in reducing the incidence and severity of BPD.

**Clinical Trial registration:**

https://www.chictr.org.cn/bin/project/edit?pid=248724, identifier ChiCTR2400091361.

## 1 Introduction

Bronchopulmonary dysplasia (BPD) is the most common morbidity in very preterm infants. The pathophysiology of the new BPD is primarily due to effects of placental dysfunction, hyperoxia, ventilator-induced lung injury, poor nutrition, abnormal blood flow, and genomic/epigenomic factors on an immature lung. Abnormal development impacts most structures of the lung including the alveoli, blood vessels, and airways, and frequently results in long-term impairment of lung mechanics and function (1, 2). Data from the Chinese Neonatal Network indicate that the primary complication among very preterm infants in China is BPD, with a prevalence rate of 29.2% (3). A cohort study in the United States involving 40, 268 newborns born at 22–32 weeks of gestation showed that the overall prevalence of BPD was 23.5% (4). Advances in medical technology, including the use of corticosteroids, endotracheal administration of pulmonary surfactant (PS), and non-invasive ventilatory support in high-risk preterm infants, have significantly reduced mortality and the risk of cerebral palsy in this population (5, 6). Despite these advancements, the prevalence of BPD has not shown significant decline (2). Inflammation plays a key role in the pathogenesis of BPD (7). Corticosteroids are widely used for the prevention and treatment of BPD due to their potent anti-inflammatory effects. However, systemic corticosteroid administration may lead to short-term and long-term adverse effects (8). Compared with systemic corticosteroids, inhaled corticosteroids offer the advantage of localized action and reduced systemic exposure, potentially maintaining therapeutic efficacy while significantly minimizing adverse effects (9). Nevertheless, there remains considerable controversy regarding their efficacy and safety.

**Figure 1:**
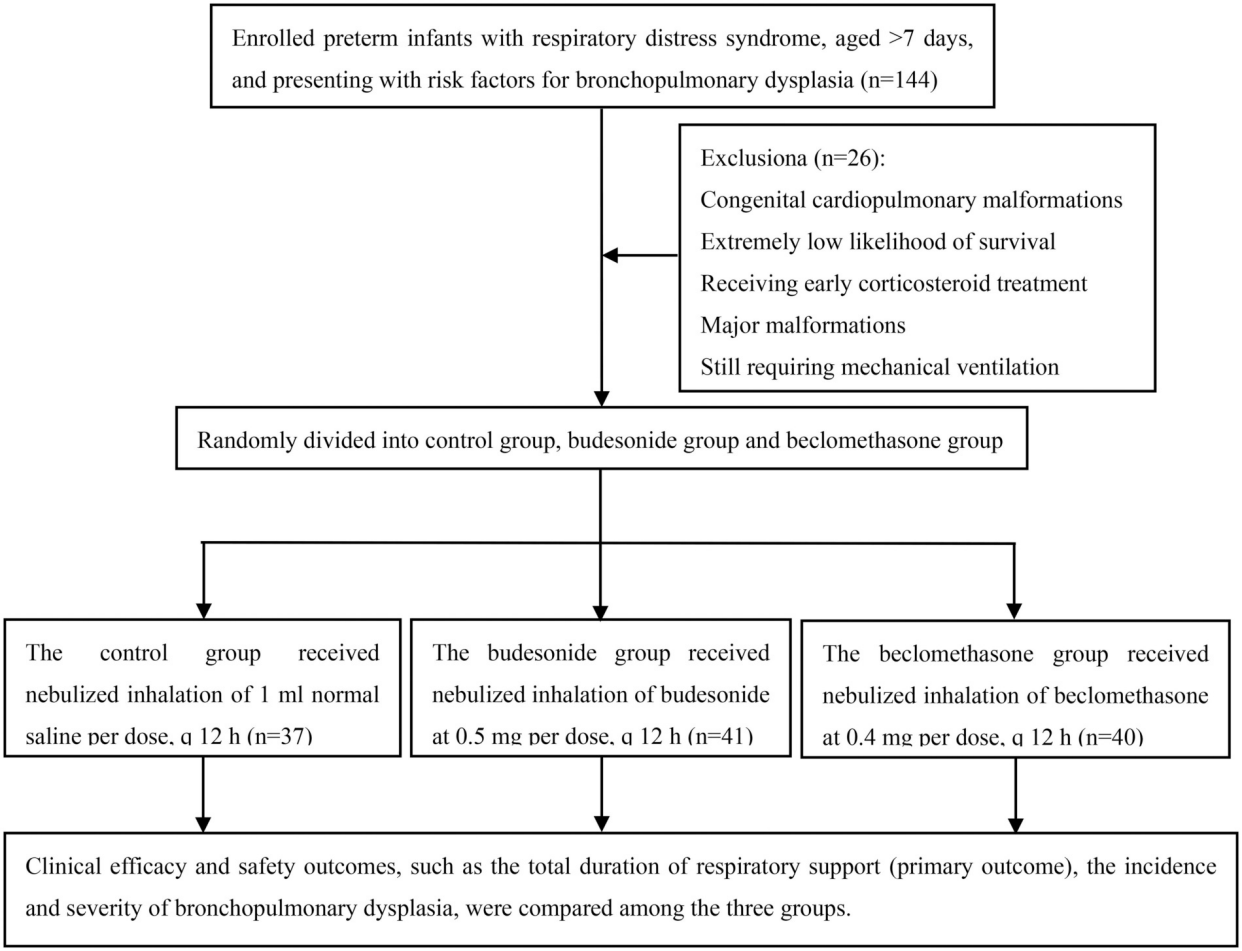
Flow diagram.

Previous studies and meta-analyses have shown that inhaled budesonide can reduce the incidence of BPD and decrease the use of systemic corticosteroids (10–13). Inhaled budesonide or beclomethasone may shorten the duration of mechanical ventilation and oxygen therapy (10). Initiating inhaled corticosteroids more than 7 days after birth does not significantly increase the risk of serious adverse events such as hypertension, sepsis, or cerebral palsy, or neurodevelopmental impairment, although the quality of evidence varies (14). Receptor affinity of beclomethasone dipropionate is higher while *in vitro* potency of budesonide is greater. Budesonide has better topical to systemic glucocorticoid activity ratio than beclomethasone dipropionate (15). Beclomethasone has traditionally been used more often for treating established BPD or respiratory complications rather than for preventive therapy (16). To maximize benefits while minimizing risks, some scholars have suggested that further research is needed to determine the optimal type, timing, dosage, and patient selection for inhaled corticosteroids in the prevention and treatment of BPD (16). Therefore, our team conducted a prospective randomized controlled study on the use of inhaled budesonide and beclomethasone for the prevention and treatment of BPD in very preterm infants, aiming to evaluate their clinical efficacy and safety and to provide a rational regimen for the clinical management of BPD in this population (Figure 1).

## 2 Patients and methods

### 2.1 Patients

This study enrolled preterm infants at high risk of BPD who were admitted to the Neonatal Intensive Care Unit of our hospital between October 2024 and November 2025. Inclusion Criteria: (i) Gestational age < 32 weeks and birth weight < 1500 g, postnatal age > 7 days. (ii) Hospital admission within 24 h of birth with a diagnosis of neonatal respiratory distress syndrome (RDS). (iii) Requiring non-invasive respiratory support and meeting at least one of the following risk factors for BPD (17–22): Requiring a fraction of inspired oxygen (FiO_2_) consistently >0.3, or gestational age < 28 weeks, or presence of hemodynamically significant patent ductus arteriosus (hsPDA), or anemia requiring blood transfusion, or oligohydramnios, or fetal growth restriction, or BPD prediction model risk score > −1.4 (19). (iv) Informed consent obtained from parents or guardians. Exclusion Criteria: (i) Congenital cardiopulmonary malformations (e.g., tetralogy of Fallot, complete transposition of the great arteries, congenital pulmonary cystic adenomatoid malformation, etc.). (ii) Infants with an extremely low likelihood of survival or for whom decisions to adjust/limit life-sustaining treatment had been made. (iii) Receiving early corticosteroid treatment for hypotension or hypoglycemia. (iv) Severe chromosomal abnormalities or major malformations (e.g., Trisomy 21, Trisomy 18, congenital tracheoesophageal fistula, congenital laryngeal atresia, etc.). (v) Clinically unstable preterm infants. (vi) Infants still requiring high-frequency or conventional mechanical ventilation. Removal Criteria: (i) Incomplete clinical data for the infant. (ii) Parental request to withdraw from the study. This study complied with medical ethics requirements and was reviewed and approved by the Medical Ethics Committee of our hospital (Approval No.: XZXY-LK-20240108-05), and was registered with the Chinese Clinical Trial Registry (ChiCTR2400091361). The guardians of all infants were informed about the treatment methods and provided signed informed consent.

### 2.2 Methods

#### 2.2.1 Data collection

After meeting the inclusion and exclusion criteria, the following demographic characteristics of the infants and potential factors associated with the occurrence of BPD were recorded: infant characteristics, sex gestational age, birth weight and its Z-score, mode of delivery, Apgar score at 5 min, use of PS, score for neonatal acute physiology-perinatal extension-Ⅱ (SNAPPE-Ⅱ) at 12–24 h after birth (perinatal supplement simplified version), hsPDA at enrollment, early-onset sepsis, ventilator-associated pneumonia (VAP); postnatal age at enrollment, risk score based on the BPD prediction model; maternal age, chorioamnionitis, antenatal steroid use, premature rupture of membranes >18 h, severe fetal distress, Ureaplasma urealyticum (UU) nucleic acid positive in the birth canal swab.

#### 2.2.2 Group-based treatment

After meeting the inclusion and exclusion criteria, patients were randomly divided into three groups using a random number table method: those with random numbers divisible by 3 were assigned to the control group, those with a remainder of 1 when divided by 3 were assigned to the budesonide group, and those with a remainder of 2 were assigned to the beclomethasone group. The control group received nebulized inhalation of 1 mL normal saline per dose, q 12 h; the budesonide group received nebulized inhalation of budesonide (AstraZeneca Pharmaceuticals, specification: 1 mg/2 mL) at 0.5 mg per dose, q 12 h; and the beclomethasone group received nebulized inhalation of beclomethasone (Chiesi Farmaceutici S.p.A., Italy, specification: 0.8 mg/2 mL) at 0.4 mg per dose, q 12 h. Peripheral venous blood was collected at enrollment and on day 14 after treatment, and after centrifugation, the supernatant was collected for measuring interleukin-8 (IL-8) and interleukin-10 (IL-10) levels using enzyme-linked immunosorbent assay. Nebulized inhalation was continued in all three groups until respiratory support was no longer required. All groups received conventional comprehensive preventive measures for BPD, including nutritional support such as adequate energy, protein, vitamin A, and measures to prevent/treat anemia; fluid restriction measures such as controlling fluid volume and sodium intake, with short-term diuretic use when necessary; respiratory support measures such as maintaining transcutaneous oxygen saturation at 0.90–0.94, utilizing non-invasive ventilation whenever possible, employing volume-guaranteed ventilation, permitting permissive hypercapnia, and administering caffeine citrate; and anti-inflammatory treatments such as short-course, low-dose dexamethasone when necessary, along with active prevention and treatment of infections.

#### 2.2.3 Clinical efficacy and safety outcomes

The primary outcome was total duration of respiratory support (including invasive ventilation, non-invasive positive pressure ventilation, and nasal cannula supplemental oxygen; including post-discharge). and secondary outcomes included incidence and severity of BPD, FiO_2_ and oxygen saturation index (OSI) within 48 h post-enrollment [OSI = (FiO_2_ × mean airway pressure [cmH_2_O])/transcutaneous arterial oxygen saturation) × 100] (FiO_2_ and OSI were recorded hourly post-enrollment, and average values were taken), levels of IL-8 and IL-10, incidence of late-onset sepsis (including fungal), hyperglycemia (fasting peripheral capillary blood glucose > 7 mmol/L), hypertension (systolic blood pressure > 80 mmHg and diastolic blood pressure > 50 mmHg in preterm infants), spontaneous intestinal perforation (SIP), necrotizing enterocolitis (NEC) (stage II–III), intraventricular hemorrhage (IVH) (grade Ⅱ–Ⅳ), incidence of retinopathy of prematurity (ROP) requiring treatment at first screening, volume of red blood cell suspension transfused, presence of hsPDA at discharge, home oxygen requirement at discharge, length of hospital stay, and hospitalization costs, among others.

#### 2.2.4 Relevant diagnostic criteria

The diagnosis was primarily based on Avery's Diseases of the Newborn (10th edition) (23). The diagnosis of BPD followed the NICHD 2018 diagnostic criteria (24).

#### 2.2.5 Sample size estimation

The primary endpoint is the total duration of respiratory support required. Based on literature (10, 11) and our preliminary trial, data on the total duration of respiratory support followed a normal distribution, with a standard deviation of approximately 15 days. It was assumed that a reduction of 10 days or more in the total duration of respiratory support in the budesonide or beclomethasone group compared to the control group would be clinically meaningful to justify the use of nebulized budesonide or beclomethasone. With a one-sided significance level of 0.05 (α = 0.05) and 80% power (β = 20%) to detect the difference between groups, and assuming a sample size ratio of approximately 1:1:1 across the three groups, the minimum required sample size per group was calculated to be 28. The calculation formula is: *n* = 2[(1.645 + 0.842)/(10/15)]^2^ ≈ 28. Given that multiple comparisons were conducted among the three groups, an additional 10% to 20% in sample size was required to control the Type I error rate compared to two-group comparisons. The adjusted sample size was approximately 28 × 1.2 ≈ 34 per group. Considering a dropout rate of no more than 10%, the initial required sample size per group was approximately 38.

### 2.3 Statistical methods

Statistical analysis was performed using IBM SPSS version 27.0. The normality of data distribution was tested using the skewness and kurtosis coefficients. Normally distributed continuous variables are expressed as mean ± standard deviation (*x¯* *±* *s*). Comparisons between two groups were conducted using independent samples or paired samples *t*-tests, while comparisons among three groups were performed using one-way ANOVA with the LSD method for *post-hoc* analysis. Non-normally distributed continuous variables are expressed as median (25th percentile, 75th percentile) [*M* (*Q1*, *Q3*)]. Comparisons between two independent groups were performed using the Wilcoxon rank-sum test (Mann–Whitney *U*-test), and comparisons among three groups were conducted using the Kruskal–Wallis *H*-test. Categorical variables are expressed as frequency (%). Comparisons between two independent groups were performed using the Chi-square test for contingency tables (Pearson Chi-square test/continuity correction Chi-square test/Fisher's exact test, as appropriate), and comparisons among three groups were conducted using the likelihood ratio Chi-square test for independent samples in R × C contingency tables. Multivariate logistic regression analysis was used to identify independent risk and protective factors for BPD in very preterm infants. *P* < 0.05 was considered statistically significant.

## 3 Results

### 3.1 General information

During the study period, a total of 247 preterm infants with “gestational age < 32 weeks, birth weight < 1,500 g, and postnatal age > 7 days” were admitted. Among them, 144 cases met the inclusion criteria. Four cases were excluded due to congenital cardiopulmonary malformations, six cases were assessed as having an extremely low likelihood of survival or had decisions made to adjust/limit life-support treatment, three cases received early glucocorticoid treatment for hypotension or hypoglycemia, three cases had severe chromosomal abnormalities, one case had congenital tracheoesophageal fistula, and five cases were extremely unstable preterm infants. Additionally, three cases were excluded due to incomplete or unreliable data, and one case was withdrawn at the parents' request. Ultimately, 37 cases were assigned to the control group, 41 to the budesonide group, and 40 to the beclomethasone group. There were no statistically significant differences (*P* > 0.05) among the three groups in terms of infant characteristics such as gender, gestational age, birth weight and its Z-score, mode of delivery, 5 min Apgar score, use of PS, SNAPPE-Ⅱ score at 12–24 h of life, hsPDA at enrollment, early-onset sepsis, VAP, postnatal age at enrollment, and BPD risk prediction model score, as well as maternal characteristics including maternal age, chorioamnionitis, prenatal steroid use, premature rupture of membranes >18 h, severe fetal distress, and positive UU in birth canal swabs (Table 1).

**Table 1:** Comparison of baseline characteristics among three groups [*n*(%)].

Variables	Control group (*n* = 37)	Budesonide group (*n* = 41)	Beclomethasone group (*n* = 40)	*χ* ^2^	*P*
Male gender	21 (56.8)	25 (61.0)	22 (55.0)	0.313	0.855
Gestation age (weeks)^a^	30.1 (29.2, 30.5)	30.4 (29.8, 31.1)	30.1 (29.0, 30.7)	4.352	0.113
Infants < 28 weeks’ gestation	3 (8.1)	2 (4.9)	2 (5.0)	10.411	0.237
Birth weight (g)^a^	1,170 (1,055, 1,275)	1,280 (1,140, 1,323)	1,260 (1,131, 1,330)	4.656	0.098
Z-score of birth weight^b^	−0.6 ± 0.7	−0.5 ± 0.6	−0.4 ± 0.6	0.611	0.545
Mode of delivery	27 (73.0)	25 (61.0)	30 (75.0)	2.186	0.335
5 min Apgar score^a^	9 (8, 9)	9 (8, 9)	9 (8, 9)	1.906	0.386
Use of PS	30 (81.1)	30 (73.2)	29 (72.5)	0.936	0.626
SNAPPE-Ⅱ score at 12–24 h of life^a^	5.0 (5.0, 12.0)	5.0 (5.0, 12.0)	5.0 (5.0, 10.8)	2.687	0.261
HsPDA at enrollment	6 (16.2)	5 (12.2)	4 (10.0)	0.684	0.710
Early-onset sepsis	4 (10.8)	7 (17.1)	7 (17.5)	0.826	0.662
VAP	1 (2.7)	0 (0.0)	2 (5.0)	1.926	0.416
Postnatal age at enrollment^b^	8.3 ± 0.5	8.3 ± 0.5	8.2 ± 0.4	0.936	0.395
BPD risk prediction model score > −1.4	12 (32.4)	15 (36.6)	15 (37.5)	0.242	0.886
Maternal age^b^	32.0 ± 5.6	29.6 ± 4.5	31.1 ± 5.1	2.222	0.113
Chorioamnionitis	5 (13.5)	4 (9.8)	3 (7.5)	0.815	0.694
Prenatal steroid use	28 (75.7)	27 (65.9)	25 (62.5)	1.637	0.441
Premature rupture of membranes > 18 h	10 (27.0)	13 (31.7)	14 (35.0)	0.571	0.752
Severe fetal distress	5 (13.5)	9 (22.0)	8 (20.0)	0.986	0.611
Positive UU in birth canal swabs	5(13.5)	3(7.3)	4(10.0)	0.868	0.643

PS, pulmonary surfactant, SNAPPE-II, score for neonatal acute physiology with perinatal extension-Ⅱ, hsPDA, hemodynamically significant patent ductus arteriosus, VAP, ventilator-associated pneumonia.

a The measurement data with nonnormal distribution were expressed by [*M* (*Q1, Q*3)], and the statistical value is the *H* value.

b The measurement data with normal distribution were expressed by (*x¯* ± *s*), and the statistical value is the *F* value.

### 3.2 Comparison of clinical efficacy, safety, and inflammatory indicators among three groups

There was a statistically significant difference in the total duration of respiratory support among the three groups of very preterm infants (*P* < 0.05). The total duration of respiratory support in both the budesonide group and the beclomethasone group were shorter than that in the control group (*P* < 0.05). There was no statistically significant difference in the total duration of respiratory support between the budesonide group and the beclomethasone group (*P* > 0.05). Similarly, there was a statistically significant difference in the length of hospital stay among the three groups (*P* < 0.05). The length of hospital stay in both the budesonide group and the beclomethasone group were shorter than that in the control group (*P* < 0.05). There was no statistically significant difference in the length of hospital stay between the budesonide group and the beclomethasone group (*P* > 0.05). There were no statistically significant difference among the three groups in the following aspects: the incidence of BPD, the incidence of mild and moderate-to-severe BPD, the average FiO_2_ and OSI within 48 h after treatment initiation, the use of systemic corticosteroids, the incidence of late-onset sepsis (including fungal infections), hyperglycemia, stage Ⅱ–Ⅲ NEC, grade Ⅱ–Ⅳ IVH, ROP requiring treatment upon first screening, volume of packed red blood cell transfusion, the presence of hsPDA at discharge, home oxygen requirement at discharge, and hospitalization costs (all *P* > 0.05) (Table 2). There were no hypertension, SIP, and deaths among the infants in any of the three groups after enrollment, including deaths within 24 h after treatment withdrawal.

**Table 2:** Comparison of clinical efficacy and safety among three groups [*n*(%)].

Variables	Control group (*n* = 37)	Budesonide group (*n* = 41)	Beclomethasone group (*n* = 40)	*χ* ^2^	*P*
The total duration of respiratory support(d)^a^	43.5 ± 13.5	36.4 ± 14.9	35.6 ± 15.1	63.784	<0.001^c^
BPD	19 (51.4)	12 (29.3)	11 (27.5)	5.866	0.053
Mild BPD	15 (40.5)	9 (22.0)	8 (20.0)	4.952	0.084
Moderate-to-severe BPD	4 (10.8)	3 (7.3)	3 (7.5)	0.474	0.843
The average FiO_2_ within 48 h^a^	0.36 ± 0.06	0.35 ± 0.05	0.36 ± 0.05	1.087	0.341
The average OSI within 48 h^a^	8.03 ± 3.08	7.34 ± 2.88	7.78 ± 3.15	0.512	0.601
The use of systemic corticosteroids	7 (18.9)	6 (14.6)	6 (16.1)	0.312	0.856
Late-onset sepsis	6 (16.2)	2 (4.9)	3 (7.5)	2.879	0.248
Hyperglycemia	15 (40.5)	14 (34.1)	18 (45.0)	1.011	0.603
Stage Ⅱ–Ⅲ NEC	2 (5.4)	1 (2.4)	0 (0.0)	2.037	0.308
Grade Ⅱ–Ⅳ IVH	4 (10.8)	5 (12.2)	4 (10.0)	0.193	1.000
ROP requiring treatment upon first screening	3 (8.1)	2 (4.9)	2 (5.0)	0.577	0.793
Volume of packed red blood cell transfusion^a^	78.0 ± 34.1	71.5 ± 32.9	74.8 ± 31.0	0.383	0.683
The presence of hsPDA at discharge	1 (2.7)	0 (0.0)	1 (2.5)	1.351	0.543
Home oxygen requirement at discharge	12 (32.4)	14 (34.1)	15 (37.5)	0.227	0.893
Length of hospital stay(d)^a^	50.4 ± 8.5	39.5 ± 10.1	39.8 ± 11.0	14.702	<0.001^d^
Hospitalization costs(ten thousand yuan)^b^	4.9 (3.0, 8.9)	4.5 (2.8, 6.4)	4.5(2.8, 7.2)	0.577	0.750

BPD, bronchopulmonary dysplasia, FiO_2_, fraction of inspired oxygen, OSI, oxygen saturation index, NEC, necrotizing enterocolitis, IVH, intraventricular hemorrhage, ROP, retinopathy of prematurity, hsPDA, hemodynamically significant patent ductus arteriosus.

a The measurement data with normal distribution were expressed by (*x¯* ± *s*), and the statistical value is the *F* value.

b The measurement data with nonnormal distribution were expressed by [*M* (*Q*1,*Q*3)], and the statistical value is the *H* value.

c The *P*-values for the comparisons between the control group and the budesonide group, the control group and the beclomethasone group, and the budesonide group and the beclomethasone group were <0.001, <0.001, and 0.286, respectively.

d The *P*-values for the comparisons between the control group and the budesonide group, the control group and the beclomethasone group, and the budesonide group and the beclomethasone group were <0.001, <0.001, and 0.915, respectively.

There were no statistically significant differences in IL-8 and IL-10 levels among the three groups before treatment (*P* > 0.05). At day 14 post-treatment, there were statistically significant differences in IL-8 and IL-10 levels among the three groups (*P* < 0.05). Both the budesonide group and the beclomethasone group showed lower IL-8 levels and higher IL-10 levels compared to the control group, with the differences being statistically significant (*P* < 0.05). However, no statistically significant difference was found between the budesonide group and the beclomethasone group (*P* > 0.05). No statistically significant differences were noted in IL-8 or IL-10 levels in the control group before and after treatment (*P* > 0.05). In contrast, both the budesonide group and the beclomethasone group exhibited significantly lower IL-8 levels and higher IL-10 levels at day 14 post-treatment compared to pre-treatment levels, with all differences being statistically significant (*P* < 0.05) (Table 3).

**Table 3:** Comparison of inflammatory indicators among three groups [*M* (*Q*1,*Q*3)].

Inflammatory indicators		Control group (*n* = 37)	Budesonide group (*n* = 41)	Beclomethasone group (*n* = 40)	*H*	*P*
IL-8 (pg/mL)	Before	178.0 (167.5, 209.5)	181.0 (169.5, 207.5)	178.5 (167.3, 217.8)	0.148	0.929
	After	179.0 (166.0, 201.0)	168.0 (157.5, 191.0)	166.5 (157.0, 207.0)	7.606	0.022^a^
*t*		−1.038	−5.615	−5.535	−1.038	−5.615
*P*		0.299	<0.001	<0.001	0.299	<0.001
IL-10 (pg/mL)	Before	18.1 (7.0, 22.3)	15.7 (7.8, 23.2)	11.5 (5.9, 22.0)	1.878	0.391
	After	18.7 (7.2, 22.7)	26.9 (19.4, 36.0)	25.1 (18.3, 34.3)	20.871	<0.001^b^
*t*		−1.604	−5.584	−5.518	−1.604	−5.584
*P*		0.109	<0.001	<0.001	0.109	<0.001

IL-8, interleukin-8; IL-10, interleukin-10.

a The *P*-values for the comparisons between the control group and the budesonide group, the control group and the beclomethasone group, and the budesonide group and the beclomethasone group were 0.012, 0.023, and 0.977, respectively.

b The *P*-values for the comparisons between the control group and the budesonide group, the control group and the beclomethasone group, and the budesonide group and the beclomethasone group were <0.001, <0.001, and 0.458, respectively.

### 3.3 Logistic regression analysis of independent influencing factors for BPD in very preterm infants

A total of 118 very preterm infants were divided into the BPD group (42 cases) and the non-BPD group (76 cases) based on the occurrence of BPD. The general characteristics, maternal conditions, and related complications were compared between the two groups of preterm infants (Table 4). Factors with *P* < 0.10 in the univariate analysis were included in the multivariate logistic regression analysis. The results indicated that “lower gestational age and lower birth weight” were independent risk factors for BPD in very preterm infants (*P* = 0.019 and *P* < 0.001, respectively), while “nebulized inhalation of budesonide or beclomethasone initiated one week after birth” was an independent protective factor (*P* = 0.049 and *P* = 0.034, respectively). After further classification and assignment, the analysis revealed: for each one-week decrease in gestational age, the risk of BPD increased by 1.060-fold (*OR*: 2.060, 95% *CI*: 1.127–3.765); for each 100 g decrease in birth weight, the risk of BPD increased by 1.236-fold (*OR*: 2.236, 95% *CI*: 1.446–3.459); compared to the control group, nebulized inhalation of budesonide initiated one week after birth reduced the risk of BPD by 60.8% (*OR*: 0.392, 95% *CI*: 0.154–0.995); nebulized inhalation of beclomethasone initiated one week after birth reduced the risk of BPD by 64.1% (*OR*: 0.359, 95% *CI*: 0.139–0.927) (Table 5).

**Table 4:** Comparison of baseline characteristics, maternal conditions and related complications between two groups [*n*(%)].

Variables	BPD Group (*n* = 42)	Non-BPD Group (*n* = 76)	*χ* ^2^	*P*
Male gender	26 (61.9)	44 (55.3)	0.489	0.485
Gestation age (weeks)^a^	29.2 ± 0.9	30.4 ± 0.9	−6.915	<0.001
Birth weight (g)^a^	1,073 ± 154	1,267 ± 102	−7.325	<0.001
Z-score of birth weight^a^	−0.6 ± 0.8	−0.4 ± 0.5	1.058	0.294
Mode of delivery	28 (66.7)	54 (71.1)	0.245	0.620
5-min Apgar score^b^	9 (8, 9)	9 (8, 9)	−1.613	0.107
Use of PS	32 (76.2)	56 (73.7)	0.090	0.765
SNAPPE-Ⅱ score at 12–24 h of life	5.0 (5.0, 12.0)	5.0 (5.0, 12.0)	−1.163	0.245
HsPDA at enrollment	9 (21.4)	6 (7.9)	3.329	0.068
Early-onset sepsis	8 (19.0)	10 (13.2)	0.726	0.394
VAP	1 (2.4)	2 (2.6)	<0.001	1.000
BPD risk prediction model score > −1.4	20 (47.6)	22 (28.9)	4.114	0.043
Maternal age^a^	31.3 ± 4.9	30.7 ± 5.2	0.603	0.548
Chorioamnionitis	5 (11.9)	7 (9.2)	0.221	0.884
Prenatal steroid use	33 (78.6)	47 (61.8)	3.468	0.063
Premature rupture of membranes > 18 h	10 (23.8)	27 (35.5)	1.725	0.189
Severe fetal distress	7 (16.7)	15 (19.7)	0.168	0.682
Positive UU in birth canal swabs	5 (11.9)	7 (9.2)	0.021	0.884
The average FiO_2_ within 48 h^a^	0.37 ± 0.06	0.35 ± 0.05	−1.330	0.186
The average OSI within 48 h^a^	8.19 ± 3.05	7.43 ± 3.00	1.313	0.192
Late-onset sepsis	5 (11.9)	6 (7.9)	0.515	0.473
Stage Ⅱ–Ⅲ NEC	0 (0.0)	3 (3.9)	—	0.552
Grade Ⅱ–Ⅳ IVH	4 (9.5)	9 (11.8)	0.006	0.938
ROP requiring treatment upon first screening	3 (7.1)	4 (5.3)	—	0.698
Volume of packed red blood cell transfusion(mL)^a^	77.7 ± 32.6	73.0 ± 32.5	−0.754	0.452
Inhaled budesonide	12 (28.6)	29 (38.2)	3.960	0.047
Inhaled beclomethasone	11(26.2)	29(38.2)	4.598	0.032

BPD, bronchopulmonary dysplasia, PS, pulmonary surfactant, SNAPPE-Ⅱ, score for neonatal acute physiology with perinatal extension-Ⅱ, hsPDA, hemodynamically significant patent ductus arteriosus, VAP, ventilator-associated pneumonia, UU, ureaplasma urealyticum, FiO_2_, fraction of inspired oxygen, OSI, oxygen saturation index, NEC, necrotizing enterocolitis, IVH, intraventricular hemorrhage, ROP, retinopathy of prematurity.

a The measurement data with normal distribution were expressed by (*x¯* ± *s*), with the statistical value being the *t*-value.

b The measurement data with nonnormal distribution were expressed by [*M* (*Q*1,*Q*3)], with the statistical value being the *Z*-value; “—” indicates the use of Fisher's exact test.

**Table 5:** Logistic regression analysis of independent influencing factors for BPD in very preterm infants.

Independent variable	Categorical assignment	B	Wald statistic	*P*	*OR* (95%*CI*)
Gestation age	<28 weeks = 4, 28–28^+^^6^ weeks = 3, 29–29^+^^6^ weeks = 2, 30–30^+^^6^ weeks = 1, 31–31^+^^6^ weeks = 0	0.723	5.522	0.019	2.060 (1.127 ∼ 3.765)
Birth weight	≤899 g = 7, 900–999 g = 6, 1,000–1,099 g = 5, 1,100–1,199 g = 4, 1,200–1,299 g = 3, 1,300–1,399 g = 2, 1,400–1,499 g = 1, ≥1,500 g = 0	0.805	13.077	<0.001	2.236 (1.446 ∼ 3.459)
Inhaled budesonide	No = 0, Yes = 1	−0.936	3.880	0.049	0.392 (0.154 ∼ 0.995)
Inhaled beclomethasone	No = 0, Yes = 1	−1.023	4.485	0.034	0.359 (0.139 ∼ 0.927)

## 4 Discussion

In the progressive stage of BPD, off-label use of inhaled corticosteroids in extremely preterm infants is a common practice (25). Inhaled corticosteroids are characterized by low systemic bioavailability, rapid systemic clearance, and high pulmonary deposition rates, thereby minimizing systemic exposure. Their theoretical advantage lies in delivering anti-inflammatory effects directly to the lungs, thereby reducing the risk of systemic adverse effects typically associated with systemic corticosteroids (16, 26, 27).

### 4.1 Nebulized inhalation of budesonide or beclomethasone shortens the total duration of respiratory support

The results of this study demonstrate that, compared with nebulized inhalation of normal saline, the use of nebulized budesonide or beclomethasone in very preterm infants who require non-invasive respiratory support one week after birth and are at risk for BPD can shorten the total duration of respiratory support and the length of hospital stay. No statistically significant differences were observed between the budesonide group and the beclomethasone group in terms of total respiratory support days or hospital stay. To investigate the effect of low-dose inhaled corticosteroids on BPD in preterm infants, Li et al. (28) included 144 preterm infants, randomly divided into a control group (*n* = 72) and a treatment group (*n* = 72). The control group received routine clinical prevention and treatment measures, while the treatment group received additional nebulized low-dose budesonide (0.5 mg per dose, twice daily) based on the control group's regimen. Both groups were treated for 10 days. The results showed that the total duration of oxygen therapy in the treatment group (23.58 ± 3.46 days) was significantly shorter than that in the control group (29.28 ± 4.43 days), with a statistically significant difference (*P* < 0.001). Zhang et al. (10) systematically reviewed 33 randomized controlled trials (RCTs) to evaluate the efficacy and safety of inhaled corticosteroids in preventing BPD in preterm infants. The meta-analysis results indicated that, compared with the control group, the budesonide group showed a significant reduction in total duration of oxygen therapy [weighted mean difference (*WMD*) −6.33 days, *P* < 0.001] and length of hospital stay (*WMD* −6.57 days, *P* < 0.001); similarly, compared with the control group, the beclomethasone group also showed significant reductions in total duration of oxygen therapy (*WMD* −10.00 days, *P* < 0.001) and length of hospital stay (*WMD* −7.06 days, *P* < 0.001). In contrast, the fluticasone propionate group showed no statistically significant differences compared with the control group in total duration of oxygen therapy (*WMD* 26.00 days, *P* = 0.376) or length of hospital stay (*WMD* −17.00 days, *P* = 0.540). Therefore, the aforementioned studies and our study suggest that nebulized inhalation of budesonide or beclomethasone can reduce the total duration of respiratory support in very preterm infants with risk factors for BPD.

### 4.2 Efficacy of nebulized budesonide or beclomethasone in the prevention and treatment of BPD

Bassler et al. (11) enrolled 863 extremely preterm infants (gestational age 23–27 ^+^ ^6^weeks) and randomly assigned them to receive either early (within 24 h after birth) inhaled budesonide or a placebo, continuing until they no longer required oxygen and positive pressure ventilation support or until they reached 32 weeks' postmenstrual age (PMA). The results showed that the incidence of BPD was 27.8% in the budesonide group compared to 38.0% in the placebo group (*RR*: 0.74, 95%*CI*: 0.60–0.91, *P* = 0.004). In extremely preterm infants, early nebulized inhalation of budesonide reduced the incidence of BPD compared with the placebo group, but was associated with a potential slight increase in mortality (16.9% vs. 13.6%; *RR*: 1.24, 95% CI: 0.91–1.69, *P* = 0.17). Delara et al. (12) conducted a systematic review and meta-analysis on the efficacy and safety of nebulized corticosteroids in preterm infants with RDS, demonstrating that nebulized corticosteroid treatment significantly reduced the incidence of the composite outcome of BPD or death (*RR*: 0.86, 95% *CI*: 0.75–0.98). Compared to the assessment of BPD at 28 days after birth (*RR*: 0.96, 95% *CI*: 0.86–1.07), the reduction in the composite outcome incidence was more pronounced and the effect stronger when assessed at 36 weeks' PMA (*RR*: 0.80, 95% *CI*: 0.68–0.94). However, the results of one studies have shown that inhaled corticosteroids did not significantly reduce the incidence of BPD (25).

In the results of our study, although multivariate logistic regression analysis indicated that nebulized inhalation of budesonide or beclomethasone initiated one week after birth was an independent protective factor against the development of BPD in very preterm infants, and although the budesonide and beclomethasone groups showed a reduced incidence and severity of BPD compared to the control group receiving nebulized normal saline, the differences were not statistically significant. This may be related to the possibility that “nebulized corticosteroids have limited efficacy in reducing the incidence of BPD”, or it could be attributed to an insufficient sample size (if BPD incidence were selected as the primary outcome, an estimated sample size of at least 200–300 cases per group would be required, which is difficult to achieve in a single-center study. Therefore, this study selected the total duration of respiratory support as the primary outcome). Additionally, variations in the gestational age, birth weight, and BPD risk factors of the preterm infants included across different studies, as well as differences in comprehensive rescue capabilities and measures, may also contribute to inconsistencies in research findings.

### 4.3 Impact of nebulized budesonide or beclomethasone on short-term respiratory support

The results of our study showed no statistically significant differences in the average FiO_2_ and OSI within 48 h after treatment initiation between the nebulized budesonide group, the beclomethasone group, and the control group. Onland et al. (14) systematically reviewed 7 randomized controlled trials (RCTs), and the meta-analysis results indicated no statistically significant difference in FiO_2_ between the nebulized corticosteroid group and the placebo group. Jónsson et al. (29) investigated the effect of nebulized budesonide on oxygen requirements in preterm infants at high risk of developing BPD. The study included 30 preterm infants with a median gestational age of 26 weeks and a median birth weight of 805 g, who were randomly assigned to group. The results revealed that although the FiO_2_ levels in the budesonide group were lower than those in the placebo group at 7, 14, and 21 days after treatment, the differences were not statistically significant. To determine the effective dose of nebulized corticosteroids in facilitating extubation or reducing FiO_2_ in ventilator-dependent preterm infants, Raghuram et al. (30) conducted a non-randomized dose-finding trial. The study included 41 very preterm infants with a gestational age < 32 weeks or birth weight < 1,250 g, who were dependent on invasive respiratory support between 10 and 28 days after birth, with a median age of 22 days. Four dose groups were studied: nebulized beclomethasone at doses of 200 μg, 400 μg, 600 μg, and 800 μg per administration, twice daily for one week. The results showed that, compared to pre-treatment levels, the average FiO_2_ within 48 h after treatment did not change significantly in the 200 μg group, while decreases were observed in the 400 μg, 600 μg, and 800 μg groups, with the greatest reduction in the 800 μg group. However, follow-up revealed that the proportion of severe cognitive impairment in the 800 μg group was slightly higher than in the other three groups. Therefore, in this study, the beclomethasone group adopted a regimen of nebulized beclomethasone at 0.4 mg per dose, administered every 12 h. The results of this study indicated that nebulized budesonide or beclomethasone did not significantly reduce short-term respiratory support intensity within 48 h, which differs slightly from the findings of Raghuram et al. (30). This discrepancy may be attributed to the fact that Raghuram et al. (30) included very preterm infants who were dependent on invasive respiratory support between 10 and 28 days after birth and had relatively more severe conditions.

### 4.4 Effect of nebulized budesonide or beclomethasone on short-term clinical outcomes

This study found no statistically significant differences among the three groups in short-term clinical outcome indicators. In the study by Bassler et al. (12), among the short-term clinical outcomes in the nebulized budesonide group, only the proportion of hsPDA requiring surgical treatment was lower than that in the placebo group, while no statistically significant differences were observed between the two groups in other short-term clinical outcomes. The meta-analysis results by Zhang et al. (10) also showed no statistically significant differences between the nebulized corticosteroid group (including budesonide, beclomethasone, or fluticasone) and the control group in the incidence of adverse outcomes. Therefore, nebulized budesonide or beclomethasone demonstrates high safety in very preterm infants.

### 4.5 Effect of nebulized budesonide or beclomethasone on inflammatory indicators

IL-8, as a serum pro-inflammatory cytokine, induces chemotactic aggregation of neutrophils and monocytes in the lungs, leading to lung injury in preterm infants. Conversely, IL-10, an anti-inflammatory factor, can inhibit the production of IL-8, thereby alleviating lung injury in preterm infants (16, 28). These two serum indicators are closely associated with the development of BPD in preterm infants (31, 32). The results of this study demonstrate that nebulized administration of either budesonide or beclomethasone can reduce IL-8 levels and elevate IL-10 levels, with no statistically significant difference observed between the budesonide and beclomethasone groups. Hagman et al. (33) conducted a prospective cohort study involving 71 very preterm infants with a median gestational age of 27.4 weeks (range 23.9–31.7 weeks). Levels of IL-8 and IL-10 were measured in umbilical cord blood and arterial blood collected at 6 h, 24 h, and 72 h after birth. The results indicated that preterm infants with BPD had significantly higher IL-8 levels and significantly lower IL-10 levels compared to those without BPD. In conjunction with the findings of this study, it is hypothesized that nebulized budesonide or beclomethasone may exert anti-inflammatory effects and contribute to the prevention and treatment of BPD by suppressing IL-8 and promoting the production of IL-10.

Limitations: (i) For very preterm infants with BPD, a rigorous follow-up mechanism must be established to clarify the risk factors leading to unfavorable trajectories of lung function development (34). Our research team is currently conducting follow-up assessments on the long-term outcomes, including neurological and physical development, of the infants in the three study groups. (ii) Future efforts should aim to identify subgroups of preterm infants most likely to benefit from nebulized corticosteroid therapy, as well as to determine the optimal timing and dosage of such treatment (16).

In summary, nebulized inhalation of budesonide and beclomethasone initiated after the first week of life can reduce the total duration of respiratory support in very preterm infants who still require non-invasive respiratory support beyond 7 days of age and present with BPD-related risk factors, without increasing the incidence of adverse outcomes and with a favorable safety profile. However, nebulized budesonide and beclomethasone did not demonstrate significant advantages in reducing the incidence or severity of BPD.

## Data Availability

The datasets presented in this study can be found in online repositories. The names of the repository/repositories and accession number(s) can be found in the article/Supplementary Material.
